# Uncovering early transcriptional regulation during adventitious root formation in *Medicago sativa*

**DOI:** 10.1186/s12870-023-04168-0

**Published:** 2023-04-04

**Authors:** Ye Ai, Xu Qian, Xiaoqian Wang, Yinglong Chen, Tiejun Zhang, Yuehui Chao, Yan Zhao

**Affiliations:** 1 School of Grassland Science, Beijing Forestry University, Beijing, 100083 China; 2 College of Life Sciences, University of Chinese Academy of Sciences, Beijing, 100049 China; 3 Beijing Tide Pharmaceutical Co., Ltd, Beijing, 100176 China; 4 The UWA Institute of Agriculture, and UWA School of Agriculture and Environment, The University of Western Australia, Perth, WA 6001 Australia; 5 College of Grassland, Resources and Environment, Inner Mongolia Agricultural University, Key Laboratory of Grassland Resources (IMAU), Ministry of Education, Hohhot, 010021 China

**Keywords:** *Medicago sativa* L., Cuttage, Adventitious Root, RNA-Seq, Differentially expressed genes

## Abstract

**Background:**

Alfalfa (*Medicago sativa* L.) as an important legume plant can quickly produce adventitious roots (ARs) to form new plants by cutting. But the regulatory mechanism of AR formation in alfalfa remains unclear.

**Results:**

To better understand the rooting process of alfalfa cuttings, plant materials from four stages, including initial separation stage (C stage), induction stage (Y stage), AR primordium formation stage (P stage) and AR maturation stage (S stage) were collected and used for RNA-Seq. Meanwhile, three candidate genes (*SAUR*, *VAN3* and *EGLC*) were selected to explore their roles in AR formation. The numbers of differentially expressed genes (DEGs) of Y-vs-C (9,724) and P-vs-Y groups (6,836) were larger than that of S-vs-P group (150), indicating highly active in the early AR formation during the complicated development process. Pathways related to cell wall and sugar metabolism, root development, cell cycle, stem cell, and protease were identified, indicating that these genes were involved in AR production. A large number of hormone-related genes associated with the formation of alfalfa ARs have also been identified, in which auxin, ABA and brassinosteroids are thought to play key regulatory roles. Comparing with TF database, it was found that AP2/ERF-ERF, bHLH, WRKY, NAC, MYB, C2H2, bZIP, GRAS played a major regulatory role in the production of ARs of alfalfa. Furthermore, three identified genes showed significant promotion effect on AR formation.

**Conclusions:**

Stimulation of stem basal cells in alfalfa by cutting induced AR production through the regulation of various hormones, transcription factors and kinases. This study provides new insights of AR formation in alfalfa and enriches gene resources in crop planting and cultivation.

**Supplementary Information:**

The online version contains supplementary material available at 10.1186/s12870-023-04168-0.

## Background

Adventitious root (AR) formation from cutting is a method of asexual propagation of plants. AR production during cutting process involves four phases: Stage 1, the stem cutting isolated from the mature plant; Stage 2 (initial phase), the initial cell reprogramming; Stage 3, primordium formation at the base of the stem cutting; Stage 4, initiation of ARs and white bulges formation [1, 2]. A large number of studies have shown differences in the ability of AR production among plant species [3, 4]. In agricultural production, rooting by cutting can not only save time, but also maintain excellent plant characters. Therefore, it is of great significance to study the molecular mechanism of ARs from cutting in agricultural production.

Auxin is an endogenous hormone containing an unsaturated aromatic ring and an acetic acid side chain, which is synthesized mainly in the young tissues of plants, such as buds and young leaves [5], and widely distributed in plant cells and tissues with vigorous growth, such as the meristem of the germ sheath, bud and root tip [6, 7], while less in tissues and organs tending to senescence. The combination of membrane diffusion and carrier-mediated transport is the mode of auxin transport between plant cells. Polar auxin transport plays a vital role in plant growth and development [8]. Auxin can regulate the growth rate of stems, inhibits the growth of lateral buds and promotes rooting [9]. It is a central player in hormone cross-talk that controls AR formation [10]. Therefore, auxin may play a critical role in promoting rooting of cuttings in agriculture. Through a mutation study of *Arabidopsis thaliana* and *Oryza sativa*, the understanding of the auxin rooting mechanism regulating plant AR production, including auxin biosynthesis, metabolism, transport, and signaling pathways, was increased [11].

In recent years, research on the regulation mechanism of AR production has made great progress at the molecular level. The use of exogenous auxin has a significant effect on the induction of ARs. Compared with indole-3-acetic acid (IAA), indole-3-butyric acid (IBA) has a better induction effect and is more widely used [10, 12]. Exogenous spermidine promotes adventitious root formation by increasing the expression of endogenous auxin and cell cycle related genes [13]. The study of Tahir, et al. [14] reveals the promoting effect of potassium by hormones and sugar signaling pathways during ARs formation in the apple rootstock. There are many studies on AR formation in woody plants and horticulture [15–18], but few studies on the regulatory mechanism of AR production in herbage.

Alfalfa (*Medicago sativa* L.) is a perennial leguminous plant, which is known as "the king of pasture" [19, 20]. As a kind of high-quality forage, alfalfa has made some progress in molecular research. Using transcriptome sequencing, Cui, et al. [21] identified alfalfa root genes related to the response to low temperature stress, which are involved in some pathways, including plant hormone signal transduction, endoplasmic reticulum protein processing, carbon metabolism, glycolysis/ gluconeogenesis, starch and sucrose metabolism, and endocytosis. Helaoui, et al. [22] revealed some alfalfa genes in response to heavy metal nickel toxicity. A study of Li, et al. [23] showed that endogenetic phages promoted the defense of alfalfa against *Acyrthosiphon pisum*, and inoculation of *Rhizophagus intraradices* increased the expression of genes related to drug resistance of alfalfa, such as "WRKY transcription factor" and "Kunitz trypsin inhibitor." However, research on AR development in alfalfa is very limited. The developmental mechanism of alfalfa ARs involving the regulation by phytohormones remains unknown. RNA-seq has become a revolutionary tool for better understanding the expression and regulatory mechanisms of differential genes. The method has no strict requirements on the reference genome sequence and is applicable to model species and non-model species [24]. Recently this technique has been used to analyze AR production in other plants including *Panax ginseng* [25], *Populus* [26] and *Camellia sinensi*s [27].

In this study, in order to understand the molecular mechanism of AR formation in alfalfa, RNA-seq was used to identify genes related to AR formation in alfalfa. Using high-throughput sequencing analysis, the changes in gene expression and the entire co-expression network during AR formation in alfalfa were monitored globally for the first time. This study not only helps to understand the regulatory mechanism of AR formation of alfalfa, but also provides new information for cutting rooting in legumes.

## Results

### Adventitious root development of alfalfa stem cutting

**Fig. 1:**
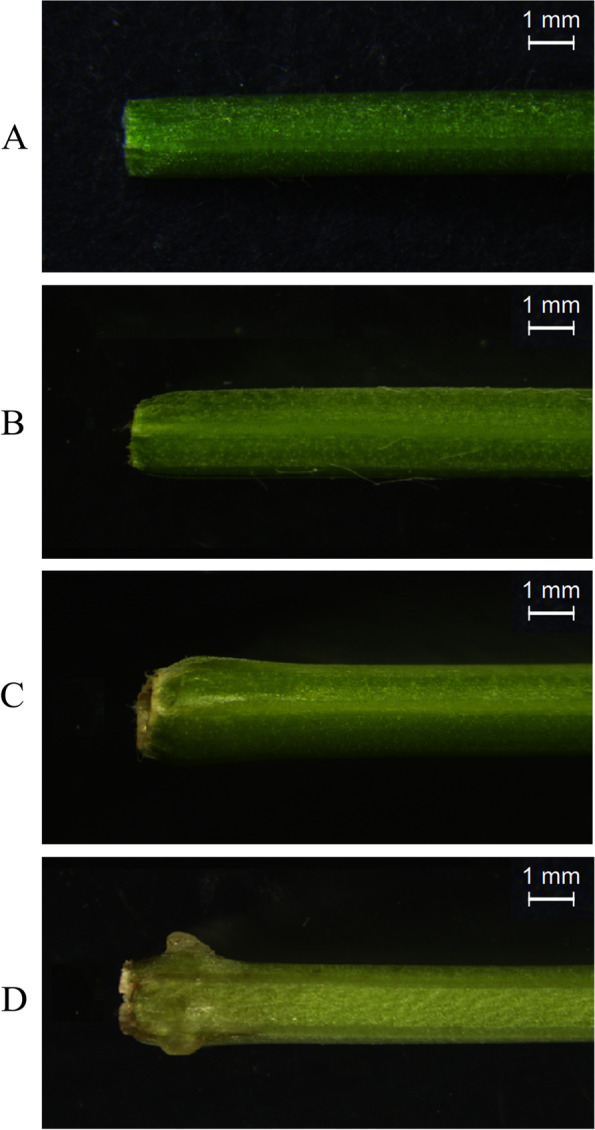
Phenotypes of four stages of adventitious root generation in alfalfa cuttings. **a** the stem cutting isolated from the mature plant (C stage); **b** the initial cell reprogramming (Y stage). **c** primordium formation at the base of the stem cutting; Stage (P stage); **d** initiation of ARs and white bulges formation (S stage)

**Fig. 2:**
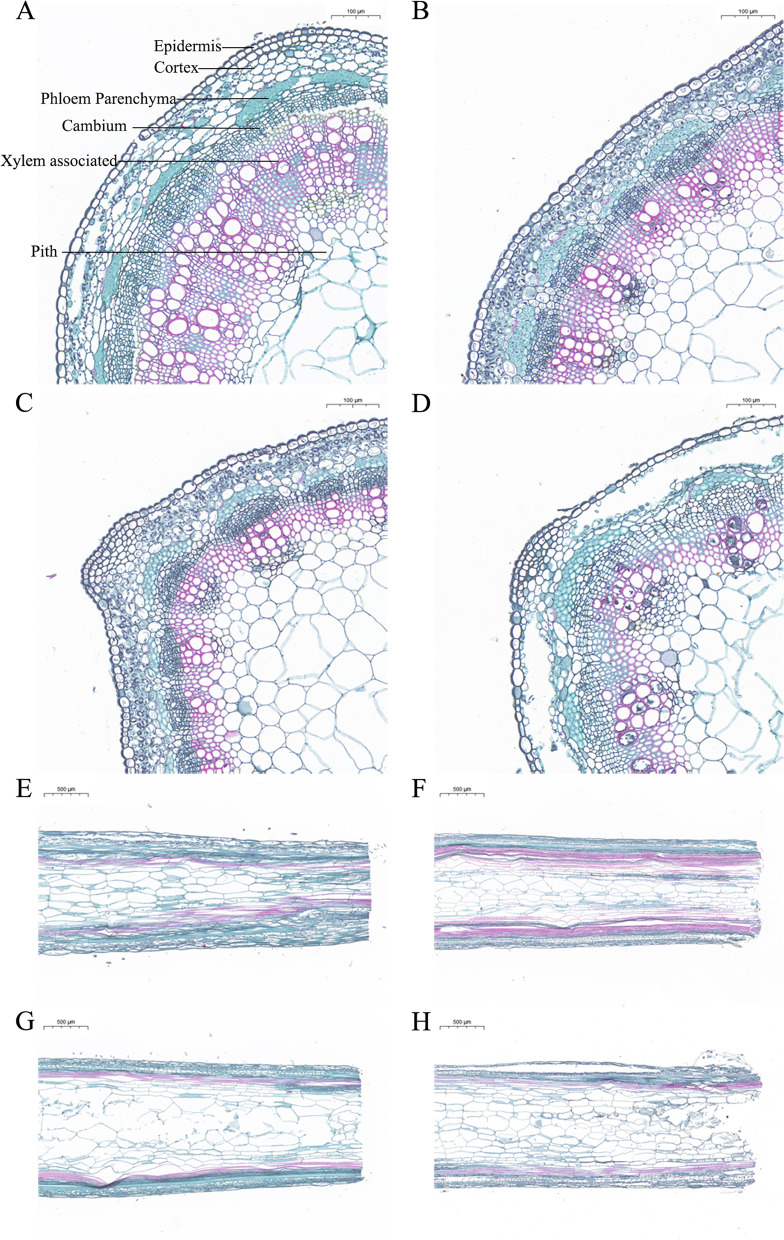
Cross sections of stem base tissue showing the development of adventitious root at different stages. **a-d** Cross section of stem base of initial separation stage (**a**), induction stage (**b**), AR primordium formation stage (**c**) and AR maturation stage (**d**). **e–h** Longitudinal section of stem base of initial separation stage (**e**), induction stage (**f**), AR primordium formation stage (**g**) and AR maturation stage (**h**). The arrows represent the direction in which the adventitious roots form

AR formation involves four phases: initial separation stage (C stage), induction stage (Y stage), AR primordium formation stage (P stage) and AR maturation stage (S stage) (Fig. 1). The section results of four stages showed that, compared with C stage, cambium cells increased and became dense, xylem and phloem were increased (Fig. 2f), the number of epidermis and cortex cells increased, but the cell size was smaller, and the number of myeloid cells decreased during the stage Y (Fig. 2a, b, e, f); Compared with Y stage, cambial cells were still in the proliferative stage, while xylem and phloem decreased, and the number of myeloid cell decreased during the stage P. At this stage, epidermal and cortical cell protrude to form AR primordium (Fig. 2c, g); Compared with P stage, Xylem and phloem decreased, the number of cambium, eidermis and cortex cells decreased but size was larger, and the number of myeloid cells increased during the stage S. At this stage, ARs protrude from the epidermis to form a white bulge, which suggests the emergence of ARs (Fig. 2d, h). Obervations revealed that ARs occurred first in xylem, phloem, cambium, epidermal and cortical, and there were significant differences in morphology of AR formation at different developmental stages.

### RNA-Seq analysis of cells in four stages of AR formation

After filtering the raw data sequenced by the Illumina platform, a total of 80.42 Gb Clean Data was obtained, including 96.72% Q20 bases with a 41.84% GC content (Table 1). The Clean Data of each sample reached 5.83 Gb. Clean Reads of each sample were aligned with reference genome ZhongmuNo.1, respectively, and the alignment efficiency was not less than 76.06% (Table S2).

**Table 1:** Data quality of RNA-Seq in *Medicago sativa*

Sample Name	Clean Reads	Clean Bases	Q20 (%)	Q30 (%)	GC Content (%)	N (%)
C1	22,528,809	6,725,027,284	98.05	94.59	42.39	0
C2	23,102,984	6,887,631,576	98.23	95.11	43.41	0
C3	24,047,730	7,167,433,678	98.37	95.42	42.28	0
Y1	21,129,771	6,338,931,300	96.79	91.36	41.84	0
Y2	21,753,381	6,526,014,300	96.72	91.23	41.95	0
Y3	22,381,492	6,714,447,600	96.79	91.41	41.79	0
P1	25,683,768	7,656,721,700	98.25	95.17	42.35	0
P2	20,890,720	6,235,774,322	96.81	92.01	41.27	0
P3	19,561,662	5,831,102,808	98.24	95.18	42.64	0
S1	24,109,775	7,201,466,086	98.27	95.19	42.15	0
S2	20,692,490	6,175,867,172	98.09	94.72	42.58	0
S3	23,324,767	6,956,505,028	98.14	94.89	43.02	0

C1-C3, the stem cutting isolated from the mature plant; Y1-Y3, the initial cell reprogramming; P1-P3, primordium formation at the base of the stem cutting; S1-S3, initiation of ARs and white bulges formation. Clean Bases, total number of bases in Clean Data. Q20, the percentage of bases with a mass value of 20 or greater in Clean Data; Q30, the percentage of bases with a mass value of 30 or greater in Clean Data; GC Content(%), the percentage of G and C bases in the total base in Clean Data. N (%), the percentage of bases with N in Clean Data

### Differentially expressed genes (DEGs) analysis of each group

**Fig. 3:**
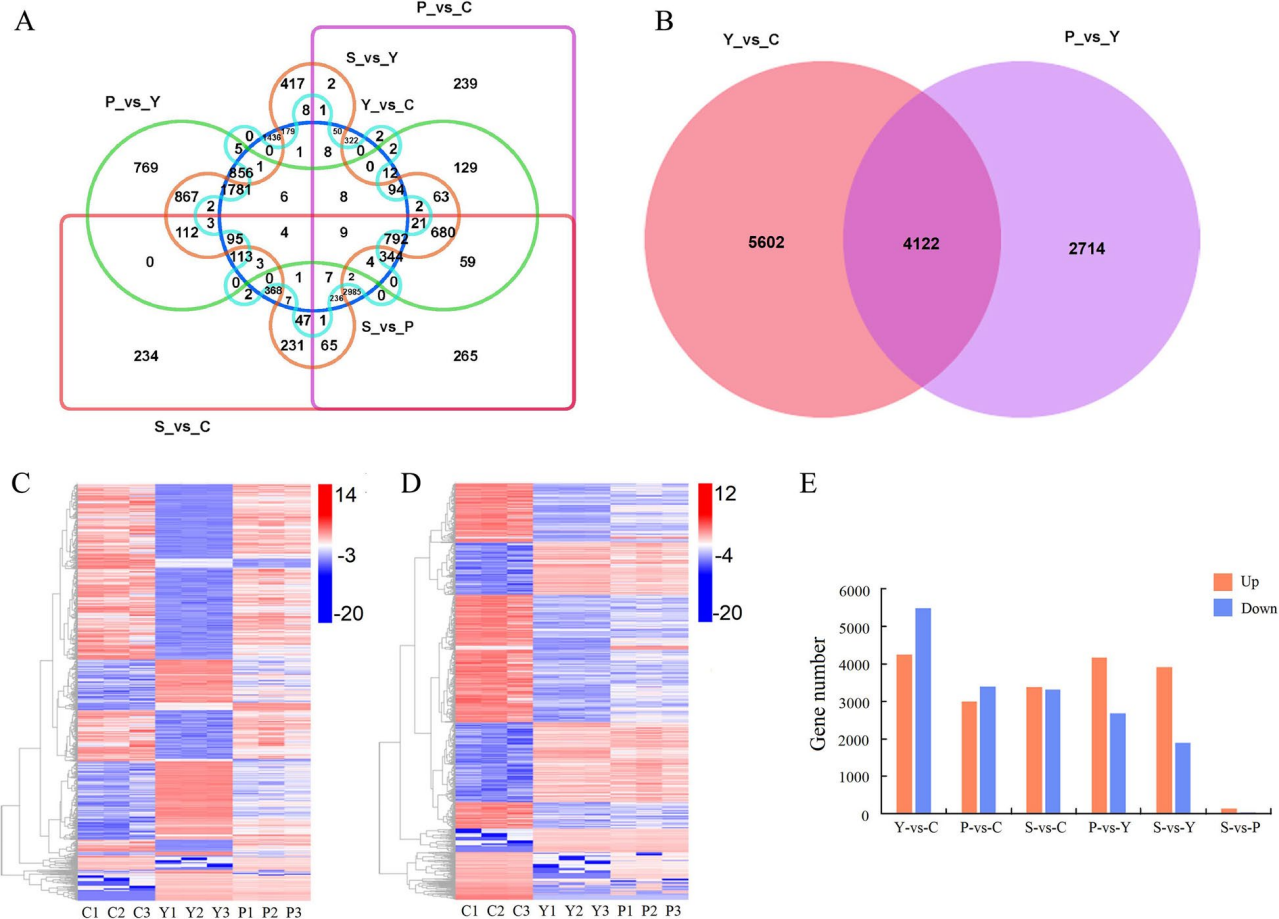
Differentially expressed genes (DEGs) identification. **a** Vern diagram of DEGs in different groups. **b** Vern diagram of DEGs in Y-vs-C and P-vs-Y groups. **c** Cluster analysis of common DEGs between Y-vs-C and P-vs-Y groups. **d** Cluster analysis of common genes between Y-vs-C and P-vs-C groups. **e** DEG number of different groups. Note: Y-vs-C: induction stage verse initial separation stage; P-vs-Y: AR primordium formation stage verse induction stage; P-vs-C: AR primordium formation stage verse initial separation stage

Differential analysis using DEseq2 software identified 9,724, 6,404, 6,690, 6,836, 5,800 and 150 genes as DEGs in the Y-vs-C, P-vs-C, S-vs-C, P-vs-Y, S-vs-Y and S-vs-P groups, respectively. The number of up-regulated DEGs were 4,241, 3,003, 3,382, 4,158, 3,909, 136, and the number of down-regulated DEGs were 5,483, 3,401, 3,308, 2,678, 1,891, 14, respectively (Fig. 3a, e). The DEG number in S-vs-P showed that there was no obvious difference between S and P stages. The number of DEGs in Y-vs-C was the largest, indicating that a large number of key genes of AR formation were transcribed during this stage. The number of DEG in P-vs-Y was 6,836, still very large, showing during Y to P formation, the regulatory genes had been drastically changed, which may be not the very early induced genes. Therefore, the C-Y and Y-P stages were analyzed as the main period of AR production.

As shown in Fig. 3b, 4,122 shared DEGs were identified in Y-vs-C and P-vs-Y groups. Interestingly, 3,839 (93%) shared DEGs in Y-vs-C and P-vs-Y groups showed opposite expression patterns (Fig. 3c). Meanwhile, there were 4,873 shared DEGs between Y-vs-C and P-vs-C groups, and 4,795 (98%) of them had the same expression trends (Fig. 3d). Those results suggested that the key genes, especially early expressed genes involved in AR formation, mainly play roles in the Y stage.

### Function annotation of DEGs

**Fig. 4:**
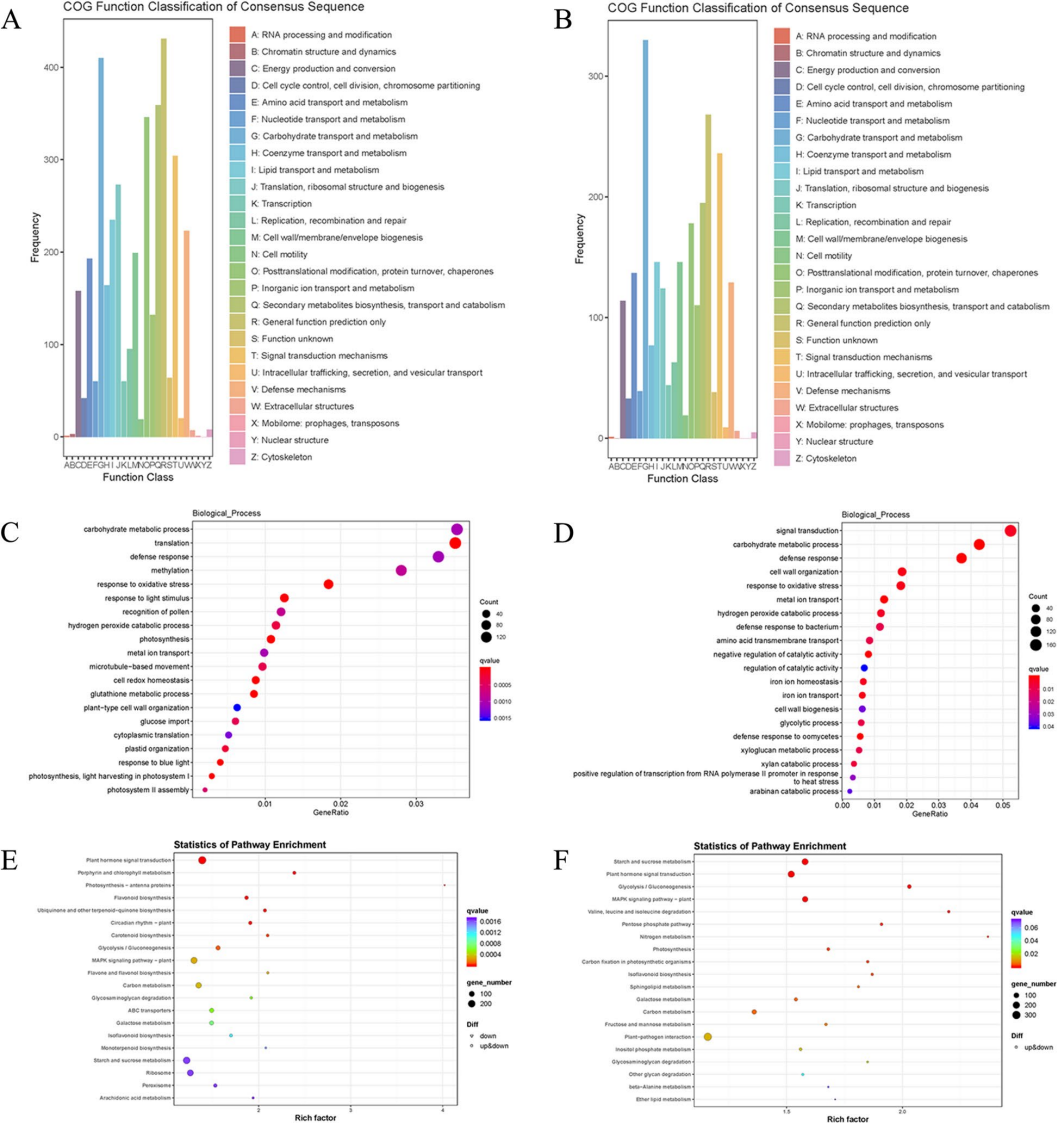
Global analysis of gene expression during AR formation in alfalfa. **a**, **b** Cluster of orthologous groups of proteins (COG) analysis of annotated DEGs in Y-vs-C (**a**) and P-vs-Y (**b**) groups, respectively. **c**, **d** Gene Ontology (GO) enrichment analysis of the annotated DEGs in Y-vs-C (**c**) and P-vs-Y (**d**) groups, respectively. **e**, **f** Kyoto Encyclopedia of Genes and Genomes (KEGG) analysis of the annotated DEGs in Y-vs-C (**e**) and P-vs-Y (**f**) groups. Note: Y-vs-C: induction stage verse initial separation stage; P-vs-Y: AR primordium formation stage verse induction stage

A total of 9,095, 6,371 and 150 DEGs were annotated in the Y-vs-C, P-vs-Y and S-vs-P groups, respectively by comparison with the NR, Swiss-Prot, KEGG, COG, KOG, GO, eggNOG and Pfam databases (Table S3). COG alignment results showed that the leading functions of DEGs in the process of AR formation included general function prediction only, carbohydrate transport and metabolism, secondary metabolites biosynthesis, transport and catabolism, posttranslational modification, protein turnover, chaperones, signal transduction mechanisms and lipid transport and metabolism (Fig. 4a, b).

### GO and KEGG analysis

#### The induction phase of AR formation (from C to Y stage)

GO analysis showed that 3,242 DEGs were enriched in Y-vs-C group. In the induction phase, 37 and 34 GO items in cell components were significantly enriched in up-regulated and down-regulated DEGs, respectively. Among them, GO items significantly enriched in up-regulated DEGs mainly include extracellular region (GO:0,005,576), ribosome (GO:0,005,840), cell wall (GO:0,005,618), microtubule (GO:0,005,874) and kinesin complex (GO:0,005,871). GO items significantly enriched in down-regulated DEGs mainly include integral component of membrane (GO:0,016,021), chloroplast (GO:0,009,507), NAD(P)H dehydrogenase complex (plastoquinone) (GO:0,010,598), photosystem I (GO:0,009,522) and photosystem II (GO:0,009,523).

There were 107 and 163 GO items in biological process significantly enriched in up-regulated and down-regulated DEGs, respectively. Among them, GO items significantly enriched in up-regulated DEGs mainly include carbohydrate metabolic process (GO:0,005,975), response to oxidative stress (GO:0,006,979), abscisic acid-activated signaling pathway (GO:0,009,738), gibberellin catabolic process (GO:0,045,487), auxin-activated signaling pathway (GO:0,009,734), microtubule-based movement (GO:0,007,018), spermine and spermidine biosynthetic process (GO:0,006,597 and GO:0,008,295) and inositol catabolic process (GO:0,009,694). GO items significantly enriched in down-regulated DEGs mainly include photosynthesis (GO:0,015,979), cell redox homeostasis (GO:0,045,454), oxylipin biosynthetic process (GO:0,031,408), chlorophyll biosynthetic process (GO:0,015,995), phloem development (GO:0,010,088), abscisic acid homeostasis (GO:1,902,265), regulation of abscisic acid-activated signaling pathway (GO:0,009,787), and sphingolipid biosynthetic process (GO:0,030,148) (Fig. 4c).

KEGG analysis on the DEGs of Y-vs-C revealed that the DEGs were enriched in 29 pathways, including plant hormone signal transduction (ko04075), porphyrin and chlorophyll metabolism (ko00860), photosynthesis-antenna proteins (ko00196), MAPK signaling pathway-plant (ko04016), carbon metabolism (ko01200), glycolysis/Gluconeogenesis (ko00010), ABC transporters (ko02010), glycosphingolipid biosynthesis-ganglio series (ko00604), peroxisome (ko04146), sphingolipid metabolism (ko00600) and so on (Fig. 4e).

#### AR primordium formation stage (from Y to P stage)

AR primordium were formed during this period. In total, 21 and 20 GO items in cell components were significantly enriched in up-regulated and down-regulated DEGs, respectively. Among them, GO items significantly enriched in up-regulated DEGs mainly include integral component of membrane (GO:0,016,021), plasma membrane (GO:0,005,886), plasmodesma (GO:0,009,506), extracellular region (GO:0,005,576) and plant-type cell wall (GO:0,009,505). GO items significantly enriched in down-regulated DEGs mainly include integral component of plasma membrane (GO:0,005,887), integral component of mitochondrial inner membrane (GO:0,031,305), chloroplast thylakoid membrane (GO:0,009,535), mitochondrial proton-transporting ATP synthase complex, coupling factor F(o) (GO:0,000,276) and small ribosomal subunit (GO:0,015,935).

There were 101 and 94 GO items in biological process significantly enriched in up-regulated and down-regulated DEGs, respectively. Among them, GO items significantly enriched in up-regulated DEGs mainly include cell wall biogenesis (GO:0,042,546), lignin catabolic process (GO:0,046,274), phloem development (GO:0,010,088), regulation of meristem growth (GO:0,010,075), regulation of salicylic acid biosynthetic process (GO:0,080,142), glycolytic process (GO:0,006,096), regulation of beta-glucan biosynthetic process (GO:0,032,951) and sterol transport (GO:0,015,918). GO items significantly enriched in down-regulated DEGs mainly include response to auxin (GO:0,009,733), photosynthetic electron transport in photosystem II (GO:0,009,772), salicylic acid metabolic process (GO:0,009,696), regulation of cysteine-type endopeptidase activity (GO:2,000,116), proline catabolic process (GO:0,006,562), jasmonic acid metabolic process (GO:0,009,694), spermine biosynthetic process (GO:0,006,597) and sphingolipid metabolic process (GO:0,006,665) (Fig. 4d).

In the P-vs-Y group, KEGG analysis revealed that the DEGs were enriched in 18 pathways, including plant hormone signal transduction (ko04075), starch and sucrose metabolism (ko00500), MAPK signaling pathway-plant (ko04016), glycolysis / Gluconeogenesis (ko00010), pentose phosphate pathway (ko00030), nitrogen metabolism (ko00910), sphingolipid metabolism (ko00600), inositol phosphate metabolism (ko00562) and so on (Fig. 4f).

### The qRT-PCR verification of DEGs from RNA-Seq

**Fig. 5:**
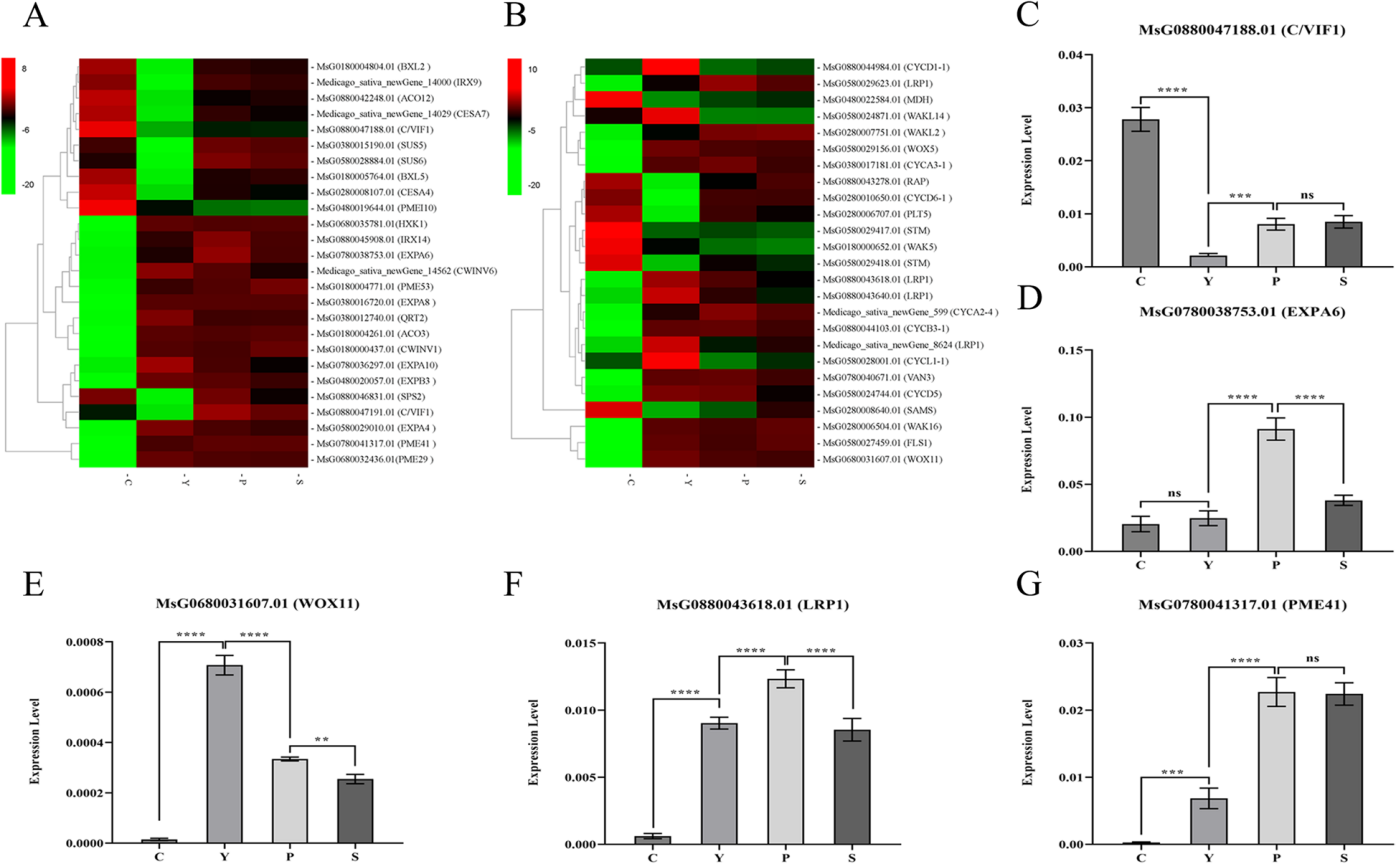
Expression profiles of DEGs in cell life cycle during alfalfa AR formation. **a** Heat map diagram of log_2_ (FPKM) values for genes annotated as cell wall degradation and remodeling and sugar metabolism related genes. **b** Heat map diagram of log_2_ (FPKM) values for genes annotated as root development-, cell cycle-, stem cell- genes and protease-related genes. **c**-**g** Relative expression of five DEGs determined by qRT-PCR. Data represent the average from four biological replicates. Data are shown as means and the error bars indicate the standard deviation (± SD). A significant difference between two stages was indicated with asterisks (ns, *, **, or ***) at ns, *P* < 0.05, 0.01, or 0.001 by Student’s *t*-test

High-throughput sequencing analysis revealed that some DEGs may play major regulatory roles in AR production. These DEGs are mainly involved in plant growth and development, mainly including *cell wall/vacuolar inhibitor of fructosidase 1* (*C/VIF*), *sucrose synthase* (*SUS*), *beta-D-xylosidase* (*BXL*), *cellulose synthase A catalytic subunit* (*CESA*), *pectinesterase* (*PME*), *sucrose-phosphate synthase* (*SPS*), *expansin* (*EXP*), *lateral root primordium 1* (*LRP1*), *cyclin* (*CYC*), *WUSCHEL-related homeobox* (*WOX*), *shoot meristemless* (*STM*) and *S-adenosylmethionine synthase* (*SAMS*) genes (Fig. 5a, b). Five genes were selected for qRT-PCR, and the results were consistent with the transcriptome results (Fig. 5c, d, e, f, g).

**Fig. 6:**
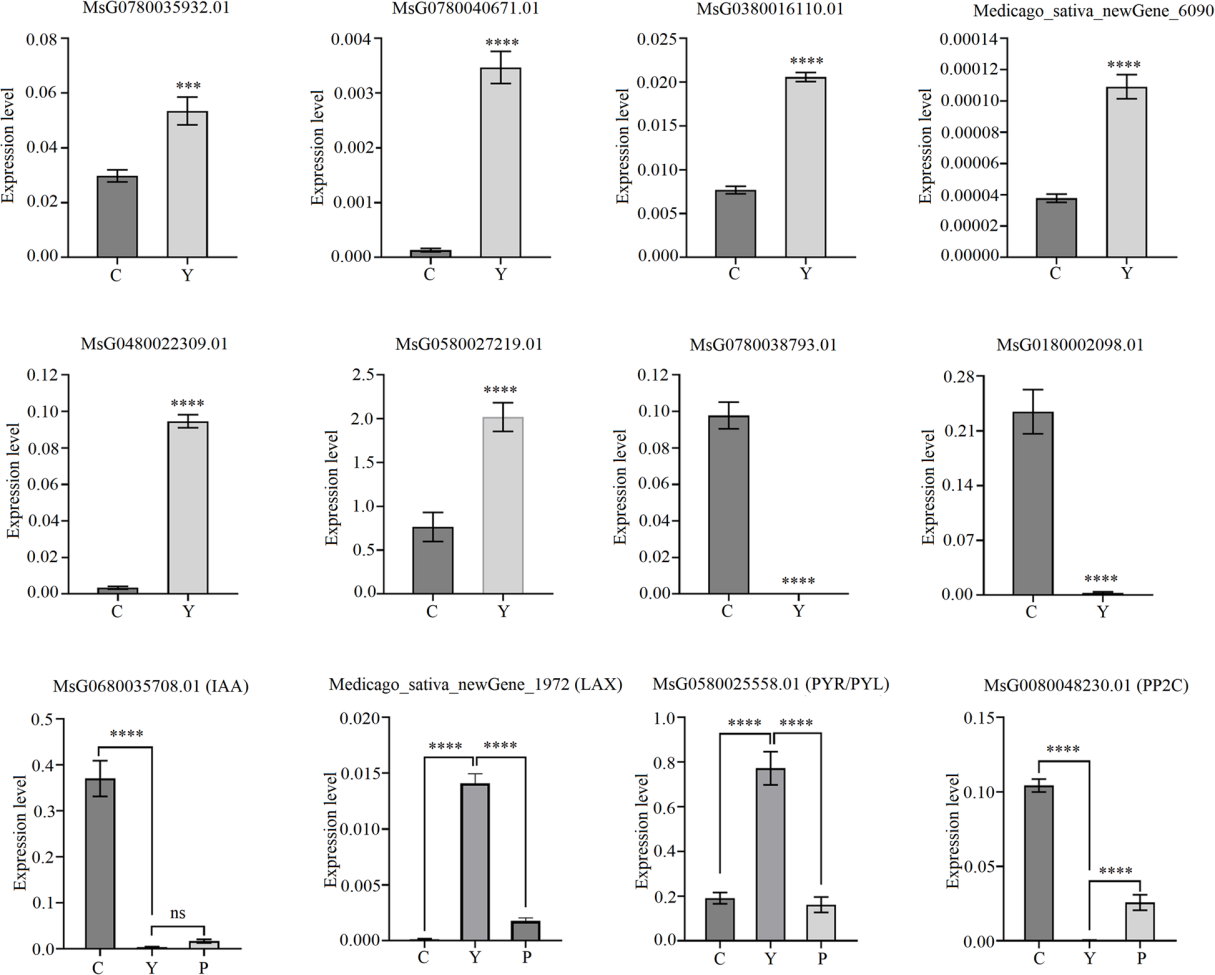
Relative expression of pathways related and hormone-related DEGs determined by qRT-PCR. Data represent the average from four biological replicates. Data are shown as means and the error bars indicate the standard deviation (± SD). A significant difference between two stages was indicated with asterisks (ns, *, **, or ***) at ns, *P* < 0.05, 0.01, or 0.001 by Student’s *t*-test

Additionally, DEGs of other pathways were also identified, such as damage repair (including ribosome and purine metabolism), ABC transporters, sphingolipid metabolism, isoflavonoid and flavonoid biosynthesis, peroxisome and so on, in Y-vs-C by RNA-Seq analysis. Therefore, it is reasonably believed that these genes were also involved in alfalfa AR production. Eight genes were selected for qRT-PCR analysis, and the results showed that their expression patterns were the same as the transcriptome results (Fig. 6a-h) (Table 2).

**Table 2:** Annotation of selected genes in induction stage compared to initial separation stage

Pathway or Category	Gene ID	Nr_annotation	log2FC
Ubiquinone and other terpenoid-quinone biosynthesis	MsG0280006579.01	Probable NAD(P)H dehydrogenase (quinone) FQR1-like 1	7.762589495
Ubiquinone and other terpenoid-quinone biosynthesis	MsG0180005984.01	Cytochrome P450 CYP73A100	4.83466062
Ubiquinone and other terpenoid-quinone biosynthesis	MsG0880044413.01	Probable aminotransferase TAT2	4.324513315
Circadian rhythm—plant	MsG0780041812.01	Protein LHY	3.46492145
Circadian rhythm—plant	MsG0580026193.01	Cyclic dof factor 5	4.103412455
ABC transporters	MsG0380013272.01	ABC transporter A family member 2	1.047581587
ABC transporters	MsG0880046436.01	ABC transporter A family member 7	1.719066661
ABC transporters	MsG0880046542.01	ABC transporter B family member 25	1.515901721
Monoterpenoid biosynthesis	MsG0580024207.01	Cytochrome P450 76T24	10.48294899
Peroxisome	MsG0180002098.01	Fe-superoxide dismutase precursor	-4.065643521
Peroxisome	Medicago_sativa_newGene_11598	Peroxisomal membrane protein PMP22	2.367295085
Peroxisome (Cutin, suberine and wax biosynthesis)	MsG0480020559.01	Fatty acyl-CoA reductase 3	-10.97791099
Peroxisome (Carbon metabolism)	MsG0280009776.01	peroxisomal (S)-2-hydroxy-acid oxidase isoform X1	-6.013934908
Phenylpropanoid biosynthesis	MsG0680035643.01	Aldehyde dehydrogenase family 2 member C4	8.474015401
Sphingolipid metabolism	Medicago_sativa_newGene_6090	Neutral ceramidase-like	8.227865291
Ribosome	MsG0280009637.01	Ribosomal protein S3	4.843228763
Ribosome	MsG0280010384.01	40S ribosomal protein S28, partial	2.7220483
Purine metabolism	MsG0380015085.01	Bifunctional bis(5'-adenosyl)-triphosphatase/adenylylsulfatase FHIT	5.73941854
Purine metabolism	MsG0280007219.01	Probable serine/threonine-protein kinase PBL23	4.641007039
Carotenoid biosynthesis	MsG0580025256.01	Probable carotenoid cleavage dioxygenase 4	-9.68501931
Carotenoid biosynthesis	MsG0080048887.01	Prolycopene isomerase	-6.471254939
Thiamine metabolism	MsG0180005666.01	Thiamine thiazole synthase	-10.38466003
Thiamine metabolism	MsG0380014728.01	Phosphomethylpyrimidine synthase	-5.21219167
Thiamine metabolism	MsG0180003780.01	MDIS1-interacting receptor like kinase 1	-4.845468687
Arachidonic acid metabolism	MsG0780041612.01	Soluble epoxide hydrolase / lipid-phosphate phosphatase	8.478870468
Fatty acid elongation	MsG0180006168.01	3-ketoacyl-CoA synthase 19	-12.61374762
Fatty acid elongation	MsG0180005969.01	3-ketoacyl-CoA synthase 6	-8.885851368
Isoflavonoid biosynthesis	MsG0180004323.01	Isoflavone 7-O-methyltransferase	9.550514169
Isoflavonoid biosynthesis	MsG0480022309.01	2-hydroxyisoflavanone synthase	6.937667505
Isoflavonoid biosynthesis	MsG0480022310.01	Isoflavone 4'-O-methyltransferase isoform X1	3.558529361
Isoflavonoid biosynthesis	MsG0480021255.01	Isoflavone reductase homolog PCBER	5.959739484
Isoflavonoid biosynthesis	MsG0280010655.01	Coumaroyl-CoA:anthocyanidin 3-O-glucoside-6''-O-coumaroyltransferase 1	-8.75598703
Flavonoid biosynthesis	MsG0780037462.01	Flavonoid 3'-monooxygenase	-10.43258405
Flavonoid biosynthesis	MsG0180006187.01	Chalcone–flavonone isomerase 2	6.133532142
Flavonoid biosynthesis	MsG0380013409.01	Flavonoid 3',5'-hydroxylase 1	8.812118138
Linoleic acid metabolism	MsG0880042650.01	Seed linoleate 9S-lipoxygenase	-12.30856399
Linoleic acid metabolism	MsG0480021111.01	Linoleate 13S-lipoxygenase 2–1	-11.11925928
Inositol phosphate metabolism	MsG0480023344.01	Inositol-3-phosphate synthase	-13.78936363
Inositol phosphate metabolism	MsG0880042494.01	Inositol oxygenase 4	11.78526427
Laccase	MsG0780038793.01	Laccase-17	-10.37844514
Laccase	MsG0380015529.01	Laccase-3	-6.480648874
Starch and sucrose metabolism	MsG0380016110.01	Glucan endo-1,3-beta-glucosidase 4	3.074206858
Steroid biosynthesis	MsG0380012835.01	Cycloartenol-C-24-methyltransferase	1.174065444

### Plant hormones that regulate AR formation

There were 350 and 297 DEGs involved in plant hormones in the Y-vs-C and P-vs-Y groups, respectively, including auxin, ABA, brassinosteroid, cytokinin, ethylene, GA, JA and SA (Table S4, S5, S6). The expression of hormone-related genes showed top three number of plant hormone-related DEGs were involved in auxin (77 and 62), brassinosteoid (67 and 50) and ABA (49 and 38) in in Y-vs-C and P-vs-Y groups, indicating that auxin, brassinosteoid and ABA play key regulatory roles in alfalfa AR formation. Four hormone-related genes were selected for qRT-PCR analysis, and the results showed that their expression patterns were the same as the transcriptome results (Fig. 6i-l).

### Transcription factors Identification

**Fig. 7:**
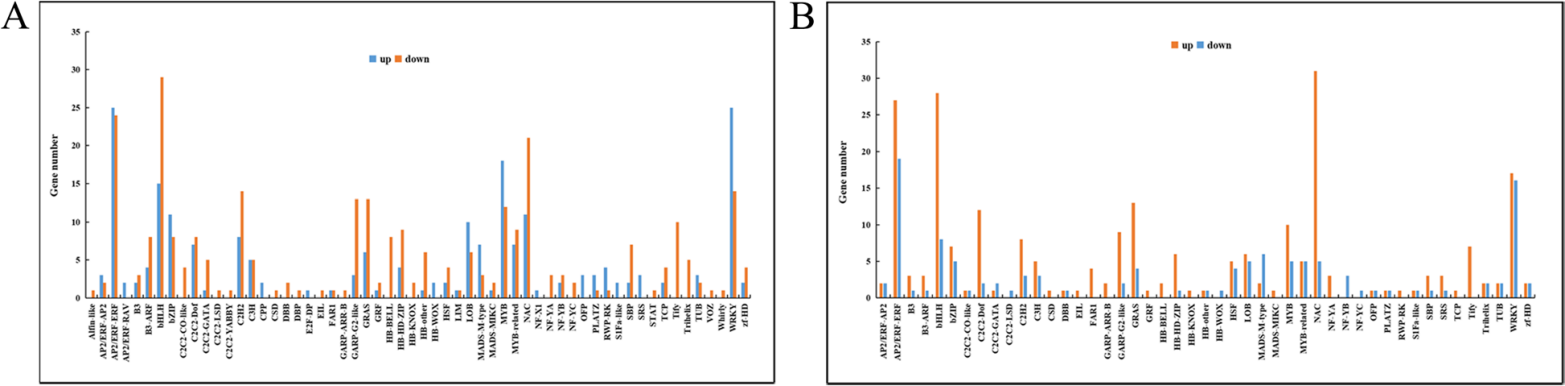
Differentially expressed factor transcripts (TFs) during adventitious root development in alfalfa cuttings. **a** TF family members were up-regulated or down-regulated in the Y-vs-C group. **b** TF family members were up-regulated or down-regulated in the P-vs-Y group. Note: Y-vs-C: induction stage verse initial separation stage; P-vs-Y: Adventitious root primordium formation stage verse induction stage

Transcription factors (TFs) revealed 501 TF genes in 58 TF families in the Y-vs-C group, among which 213 were up-regulated and 288 were down-regulated (Fig. 7a), and the top 8 families were AP2/ERF-ERF (49), bHLH (44), WRKY (39), NAC (32), MYB (30), C2H2 (22), bZIP (19), GRAS (19). In the P-vs-Y group, 362 TFs were differentially expressed, of which 243 were up-regulated and 119 were down-regulated, and the main 8 families were AP2/ERF-ERF (46), bHLH (36), NAC (36), WRKY (33), GRAS (17), MYB (15), C2C2-Dof (14), C2H2 (11) (Fig. 7b). In the S-vs-P group, there are only 11 differentially expressed TFs. Notably, bHLH, C2H2, GRAS, NAC, GARP-ARR-B, GARP-G2-like and C2C2-Dof were mainly down-regulated in the Y-vs-C group, and on the contrary, they were mainly up-regulated in the P-vs-Y group. These TFs were first down-regulated and then up-regulated in AR formation stage, resulting in a series of changes in the expression of downstream genes. It is concluded that these TFs play an important role in the regulation of AR production in alfalfa.

### Function identification of candidate genes

**Fig. 8:**
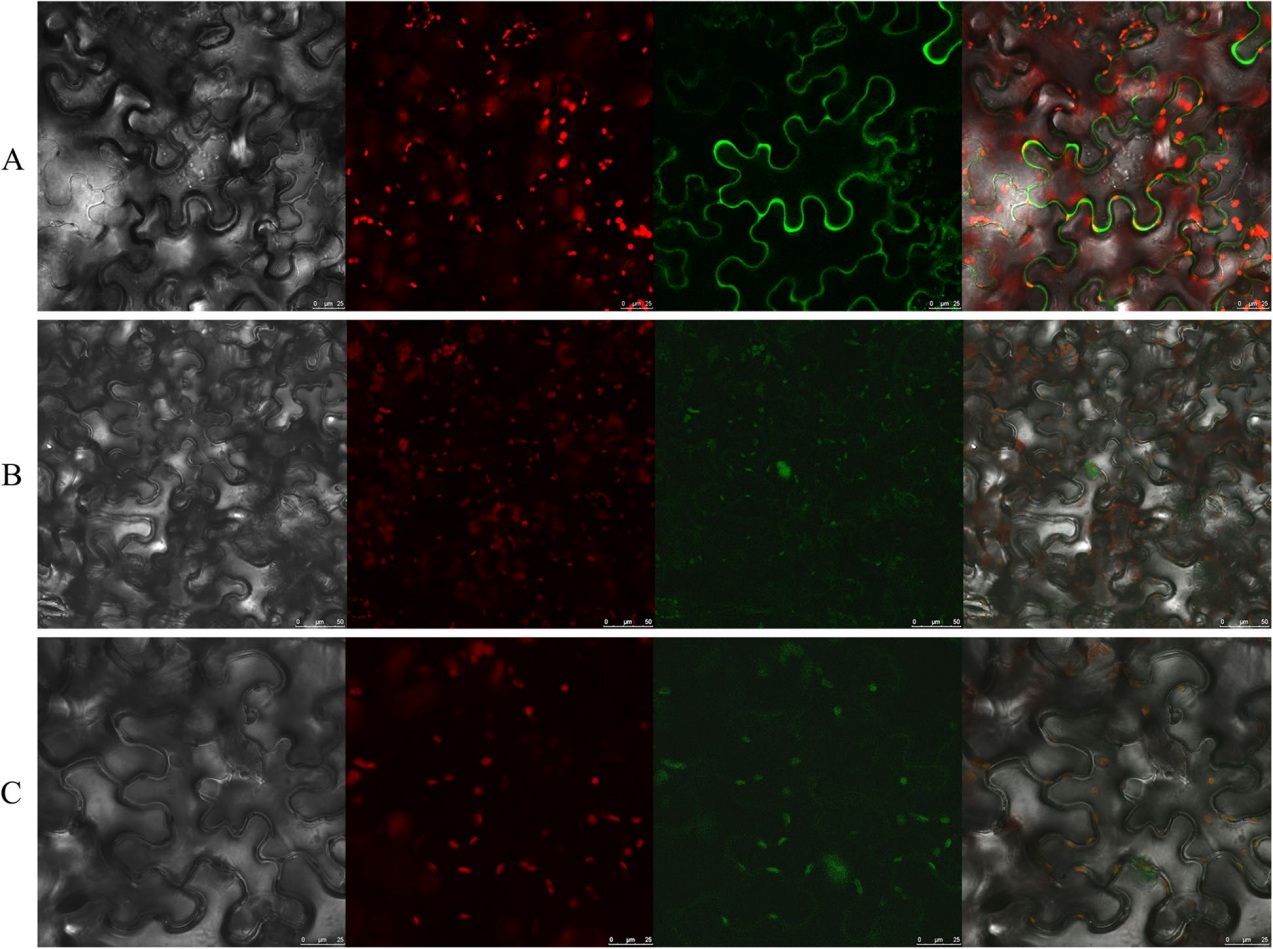
Subcellular mapping of three candidate genes. **a-c** MsG0780035932.01 (**a**), MsG0780040671.01 (**b**) and MsG0380016110.01 (**c**) is localized in cell membranes, chloroplasts and chloroplasts, respectively

RNA-Seq and qRT-PCR results showed that the expression levels of MsG0780035932.01 (*SAUR*), MsG0780040671.01 (*VAN3*) and MsG0380016110.01 (*EGLC*) were significantly up-regulated in Y-vs-C group (Fig. 6a-c), which were selected to explore the effect on AR formation. Subcellular localization analysis showed that MsG0780035932.01, MsG0780040671.01 and MsG0380016110.01 were localized in cell membrane, chloroplast and chloroplast, respectively (Fig. 8a-c).

**Fig. 9:**
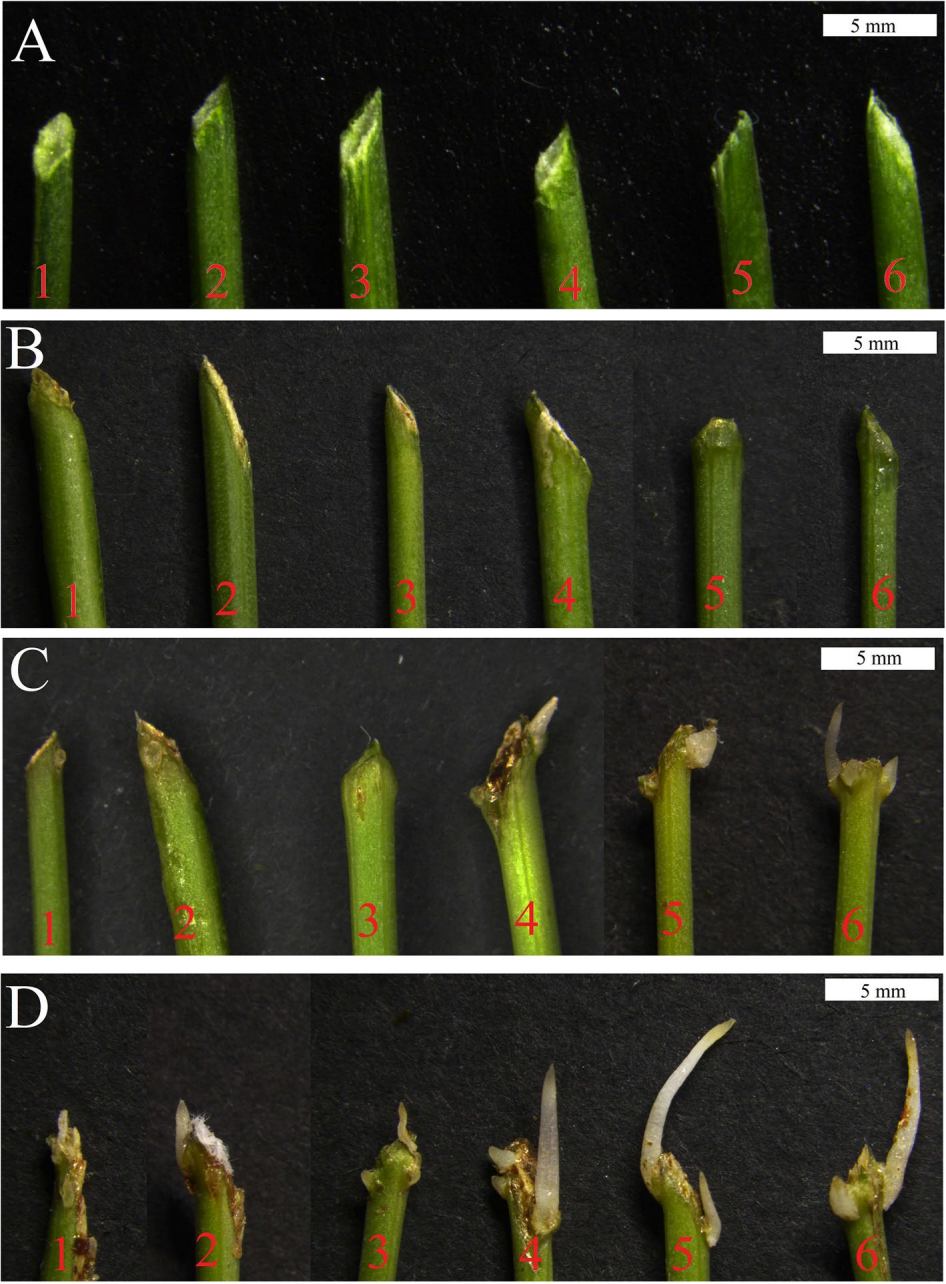
The phenotypes of alfalfa branch segment with different candidate genes. **a** On day 0, the base of both the control group and experimental group was the stem state. **b** Four days later, the base of the water treatment in the control group was enlarged. The other two treatments in the control group showed no change, while the base of the three treatment groups in the experimental group was enlarged. **c** Eight days later, the base of the control group was enlarged, while the adventitious roots of the experimental group were basically formed. **d** On the 13th day, the adventitious roots of the control group had just grown, while adventitious roots of the experimental group were significantly longer than those of the control group. Note: 1–6 water, EHA105, no-load, MsG0780035932.01, MsG0780040671.01 and MsG0380016110.01

Transformation of three genes was performed in alfalfa cuttages, respectively. Four days after co-culture, the base of alfalfa branch segments in transgenic samples swelled. However, there was no obvious change in controls (Fig. 9a, b). Eight days later, ARs were basically formed at the base of branch segments of three transgenic samples, while the bases in controls were at the starting to enlarging stages (Fig. 9c). Thirteen days later, the ARs of controls had just been observed, but the AR lengths in transgenic groups were much longer (Fig. 9d), which indicated the three genes played significant roles in promoting early initiation and formation of AR in alfalfa (Table 3).

**Table 3:** Length of adventitious roots at the base of alfalfa stems

	Sample					
Time	Control 1 (cm)	Control 2 (cm)	Control 3 (cm)	Experimental 4 (cm)	Experimental 5 (cm)	Experimental 6 (cm)
Day 8	0	0	0	1.33	2.58	2.17
	0	0	0	1.58	3.70	2.26
	0	0	0	1.59	2.25	1.91
Day13	1.26	2.31	1.95	7.38	8.20	7.98
	0.92	1.51	1.36	8.31	13.4	10.80
	1.64	1.89	2.12	7.66	9.56	8.26

The alfalfa branch segment was treated with water, agrobacterium EHA105, no-load, MsG0780035932.01, MsG0780040671.01 and MsG0380016110.01 (the first three are the control 1, 2, 3 and the last three are the experimental 4, 5, 6)

## Discussion

AR production is a complex process of plant regeneration [28]. Studies on AR formation have been reported in many plant species with a focus on hormone-induced AR production, and single or multiple genes related to AR formation [29, 30]. In this study, alfalfa cuttings at four AR formation periods were sampled for RNA-Seq analysis, resulting in 80.42 Gb of Clean Data and a large number of DEGs involved in AR formation. Meanwhile, three candidate genes from RNA-Seq were confirmed as positive regulators in AR production.

### The location of AR production in alfalfa stem

The location of AR formation depends on the species or organ or tissue. The sites where root primordium can occur include cortex, phloem, cambium, medullary rays, xylem and callus. The types of AR formation can be divided into cutaneous rooting, callus rooting and mixed rooting according to where the root primordium is produced [31–33]. In Arabidopsis, ARs emerged from the mesocotyl sheath of the plant hypocotyl [34, 35]. In apples (*Malus pumila*), AR primordium raised from interfascicular cambium cells closest to phloem cells [36]. AR primordium of *Pistacia chinensis Bunge* originated from callus, medullary ray, xylem, vascular cambium, secondary phloem, cortex, and the junction of medullary ray and cambium, which belonged to multi-locus occurrence mode and multi-type rooting mode [37]. The present study showed that the way of alfalfa ARs formation by cutting in nutrition soil was cutaneous rooting without callus, and ARs firstly occurred in xylem, phloem, cambium, epidermal and cortical at the stem base.

### The period before AR primordium production in alfalfa is an active molecular regulatory process

The results showed that there were four key nodes in the alfalfa AR production. The results that the stem base tissues of four stages of alfalfa were sliced and stained showed that cells at the base of the stem have been changing in response to stimuli and environmental changes since cutting. Therefore, it was speculated that cutting was the most direct and effective stimulation method for alfalfa AR production. The rapid response of stem basal cells to stimulation leads to a dynamic process of cell rearrangement rather than a sequential process. In the induction stage, the internal cells are constantly increasing and accumulating, which is a quantitative accumulation process, although no apparent changes was observed in the stem base morphology. Compared with the two later stages, the most DEGs (9,724) were identified in the induction stage, and the cells were rearranged at this stage. Therefore, the induction period was considered as the key stage to promote the AR production of alfalfa. In the AR primordium formation stage, 6,836 DEGs were identified, while only 150 DEGs were obtained in AR maturation stage. Therefore, AR primordium formation is the critical stage of the successful generation of ARs.

Both sugars and auxin play important roles in AR production, but higher concentration of sucrose or IAA could inhibit AR production by promoting the synthesis of laccase family LAC17 in the polymeric lignin metabolism pathway [38, 39]. In this study, MsG0780038793.01 (*LAC17*) was significantly down-regulated during the induction phase (Fig. 6g), suggesting that the reduction of polymeric lignin was beneficial to the AR production in alfalfa. In this study, In the induction stage, *PME53*, *PME29*, *PME41*, *EXPA8*, *EXPA4*, *EXPA6*, *EXPA10*, *EXPB3* were all up-regulated (Fig. 5a, d, g). over expressions of *PME* and *EXP* genes involved in cell wall degradation and remodeling were also observed in pepper (*Capsicum annuum* L.) hypocotyl found [40]. Several studies have shown that WUS plays an important role in totipotent embryonic stem cells [41] and AR production [42]. *WOX* gene can regulate AR development by regulating hormone transduction related genes, such as *ARF*, and root development related genes, such as *CYCD3*. The study of Liu and Xu [43] found that *CrWOXA* gene was specifically expressed in root founding cells during AR initiation in fern *Ceratopteris richardii*. In *Arabidopsis thaliana*, the expression switch from *WOX11* to *WOX5* is essential for AR initiation [44]. In this study, *WOX11* were significantly up-regulated (Fig. 5a, e), and a large number of genes related to hormone signal transduction and root development were identified in the induction stage. Therefore, it is believed that the expression of *WOX* could induce AR production.

The accumulation of proteins related to dephosphorylation, glycolysis and tricarboxylic acid cycle pathways and the reduction of isoflavone metabolic proteins were associated with the promotion of AR initiation [45]. The present study illustrated that protein phosphorylation, glycolysis and isoflavone metabolism have important effects on the production of alfalfa ARs. Polyamines are a class of small molecular compounds containing multiple amino groups in organisms, mainly including putrescine, spermidine and spermine, which can promote cell division and differentiation [46]. Spermine could significantly improve the effects of Lingfasu on inducing the formation of ARs of *Siraitia grosvenori* [47]. And Spermidine promoted the AR formation of apple rootstock by enhancing the expression of spermidine and auxin biosynthesis related genes [13]. The present study indicated that the increase of spermine and spermidine biosynthesis could promote the production of alfalfa ARs.

### Identification of Hormonal Signal Transduction during AR formation in Alfalfa

Phytohormones play a key role in AR formation. Numerous studies have confirmed that auxin plays a central role in AR formation [48]. Auxin influx carrier AUX/LAX and PIN families encode auxin input and export carrier proteins, respectively [49–51], which both mediate the polar transport of auxin. *DIAGEOTROPICA* encodes a cyclophilin A-type protein (SlCYP1), which regulates auxin polar transport by altering the abundance of PIN at the plasma membrane, thereby positively regulating the initiation of ARs [52–54]. In this study, PIN also plays an important role in the initiation of alfalfa ARs. TIR1 is an auxin receptor mediating transcriptional responses to auxin [55]. The IAA-amino acid ILR encodes an IAA-amino acid hydrolase [56, 57]. This study found that LAX, PIN, TIR1, AUX/IAA, ARF, GH3 and SAUR were involved in auxin signal transduction. The ARF family is one of the basic modules of AR initiation [58]. Gutierrez, et al. [59] demonstrated that the ARF transcription factor regulatory network controls initiation of ARs by regulating *GH3* gene that are required for AR initiation in Arabidopsis hypocotyl. Meanwhile, Lakehal, et al. [60] believed that TIR1 and AFB2 proteins interact with IAA to control AR initiation in *Arabidopsis Thaliana*. In the induction phase of AR production, *LAX*, *ILR and ARF* were mainly down-regulated. *SAUR*, *PIN and GH3* were mainly up-regulated. In the root primordium formation stage, *LAX*, *PIN*, *TIR1*, *ILR* and *ARF* were mainly up-regulated, and *SAUR* was mainly down-regulated. Therefore, it is concluded that the production of alfalfa ARs is achieved through two pathways: IAA transport, distribution and IAA signal response. And *LAX* and *PIN* play important regulatory roles in the formation of ARs.

PYR/PYL family protein is a receptor of ABA, which can bind to ABA to inhibit the activity of PP2Cs [61, 62]. The lack of PP2Cs phosphatase inhibition makes SnRK2 in a phosphorylated active state, resulting in activating downstream TFs ABF/AREB and positively regulating the expression of ABA signaling response genes [63–65]. In this study, in the induction stage, MsG0580025558.01 (*PYL1*) was significantly up-regulated and MsG0080048230.01 (*PP2C*) was significantly down-regulated (Fig. 6k, l), which positively regulated ABA signal transduction. Instead, MsG0580025558.01 (*PYL1*), was significantly down-regulated, and MsG0080048230.01 (*PP2C*) were significantly up-regulated in AR primordium formation, indicating that ABA signal transduction was inhibited. ABA plays an important role in abiotic stress [66]. The activation of ABA signal transduction during the early stage of AR production may be related to the response to external stress.

Brassinolides (BRs) play a role in promoting plant cell elongation, cell division, plant light morphogenesis, plant yield, and enhancing plant tolerance to environmental stress [67]. Brassinolide biosynthesis is catalyzed by cytochrome P450, including CYP90B, CYP90A1, CYP92A6, CYP85A1 and CYP734A1 [68, 69]. *CYP734A1* encodes a photochrome B activator marker inhibitor that inactivates BR [70, 71]. In the induction stage of alfalfa AR formation, *CYP90A1* and *CYP92A6* were significantly down-regulated, while *CYP734A1* was significantly up-regulated, indicating that brassinolide biosynthesis was inhibited. In root primordium formation, *CYP92A6* and *CYP85A1* were up-regulated, which promoted brassinolide biosynthesis. BRs regulates the expression of downstream *CYCD3* and *TCH4* through signaling pathways transduction, *CYCD3* mainly regulates cell division [72], and *TCH4*, encodes Xyloglucan endotransglycosylase (XET) enzymes, mainly regulates cell elongation [73]. In the induction phase, BRs promote cell division mainly through the unique CYCD3 induction pathway, and reduce cell elongation by down-regulating the expression of *TCH4*. However, in the root primordium production, BRs promoted cell elongation by inducing the expression of *TCH4*, which may be one of the reasons for the expansion of the stem base. These results demonstrated that BR promoted the production of alfalfa ARs mainly by regulating cell division and elongation (Fig. S2).

### TFs involved in AR production of alfalfa

In this study, 501 and 362 TFs were differentially expressed in Y group and P-vs-Y groups, respectively. TFs play a crucial role in regulating AR formation in alfalfa. ERFs can regulate ethylene response genes, whose expression responds to ethylene and a variety of extracellular stimuli, including abiotic stress, thereby affecting the formation of ARs [74]. Trupiano, et al. [75] found that the formation of ARs of *Populus* is controlled by the AP2/ERF family. In this study, the ERF accounted for the largest proportion in the two groups of TFs, respectively. Zhang [40] found that the expression of 12 *CaGRAS* genes was induced in the process of hypocotyl AR development of pepper. *CaGRAS1*, *CaGRAS2*, *CaGRAS21*, *CaGRAS34* and *CaGRAS49* may be important regulators of AR formation. In this study, 19 and 17 *GRAS* were identified in the Y-vs-C and P-vs-Y groups, respectively, indicating that ERF and GRAS play important regulatory roles in the AR formation of alfalfa.

The results of Mao, et al. [76] showed that overexpression of *MsWRKY100* could significantly improve the root length and drought resistance of transgenic *Arabidopsis thaliana* by reducing the malondialdehyde content and ion leakage rate, significantly increasing the activities of antioxidant enzymes and the sensitivity of Arabidopsis seedlings to abscisic acid. The relative expression of *WRKY* was significantly up-regulated during AR formation in control apple rootstock [77]. In this study, 25 *WRKY* were up-regulated and 14 were down-regulated, which was consistent with the results of [77]. It is demonstrated that the up-regulation of *WRKY* may alleviate the water shortage and promote the formation of alfala ARs. Therefore, the TFs among DEGs may be the key factor triggering the generation of ARs.

### AR production promoting genes

Three DEGs were selected for transformation and rooting tests. As one of the three auxin early response genes, *SAUR* has positive effects on cell expansion, shade avoidance response, high-temperature induced growth, tip development, leaf growth, and auxin transport during plant growth and development [78–80]. In Arabidopsis, overexpression of *AtSAUR19* results in hypocotyl elongation and increased leaf area. Overexpression of *AtSAUR41* significantly increased hypocotyl epidermal cells [81]. In this study, MsG0780035932.01, significantly up-regulated in the induction phase (Fig. 6a), could promote the production of ARs and accelerate hypocotyl elongation during the cutting process of alfalfa, which is basically consistent with the above research results.

*VAN3,* encoding an adenosine diphosphate (ADP)-ribosylation factor-guanosine triphosphatase (GTPase)-activating protein (ARF–GAP), not only plays an important role in the construction of the vascular system, but also is related to auxin signaling and polar transport. The auxin-dependent induction of *VAN3* may be positively regulated by *VAN3* itself [82]. In this study, MsG0780040671.01 gene (Fig. 6b) promoted the production and elongation of ARs, and the effect was the most obvious, which may be related to the induction of auxin.

MsG0380016110.01, encoding Glucan endo-1, 3-beta-glucosidase 4, was also up-regulated (Fig. 6c) during induction stage. β-glucosidase can promote the decomposition of isoflavone glycosides [83], and the decrease of isoflavone metabolic protein accumulation is related to the promotion of AR initiation [45]. Therefore, it is speculated that MsG0380016110.01 could induce AR production by regulating isoflavone metabolism. In addition, β-glucosidase also has effect on activating plant hormones. The study of [84] found that β-glucosidase could produce ABA by mediating the hydrolysis of Glc-conjugated ABA (ABA-GE) and play roles in osmotic stress reactions. Brzobohaty et al. [85] found that β-glucosidase was located in the meristem of maize roots and could release active cytokinins. Therefore, it is speculated that MsG0380016110.01 may also promote the production of ARs by activating hormones. The regulatory mechanisms of the three genes need to be further studied.

In general, many genes are involved in different biological processes and pathways during the rooting of alfalfa. And candidate genes such as root development-, cell cycle-, TFs-, and hormone-related genes were identified in AR formation. This study enhances our understanding of the regulatory mechanism of rooting in alfalfa cuttings, and these findings provide a preliminary functional genomics framework for understanding the complexity of alfalfa cutting rooting, which is conducive to the application of cuttings in production.

## Conclusions

Compared with S-vs-P group, there were more DEGs in Y-vs-C (9,724) and P-vs-Y groups (6,836), and the qRT-PCR results were consistent with the transcriptional sequencing results. Cluster analysis showed that there were 4,122 identical genes between the Y-vs-C and P-vs-Y groups, and 3,839 (93%) had the opposite expression trend. There were 4,873 identical genes between Y-vs-C and P-vs-C groups including 4,795 (98%) had the same trend in expression. Thus, Y stage is the key stage of alfalfa AR formation. Many genes and pathways related to AR production of alfalfa were identified by GO and KEGG enrichment analysis. Meanwhile, various hormones and TFs also play vital roles in regulating AR formation, Three DEGs were selected for transient transformation of alfalfa stem base, the result showed that they all promoted the generation and elongation of ARs in alfalfa cutting, but their regulatory mechanisms need to be further studied. In conclusion, the outcomes of this study enhances our understanding of the regulatory mechanism of AR formation of alfalfa cuttings.

## Methods

### Plant materials and growth conditions

Seeds of alfalfa (*Medicago sativa* L.) ‘Zhongmu No.1’ obtained from the Chinese Academy of Agricultural Sciences, Beijing, China were sown and cultured in a greenhouse with 16 h of light, 25 ℃ of temperature, 8 h of darkness, 23 ℃ of temperature, 14,000 lx of light intensity, 35–60% of air humidity, and watered every three days. After three months, shoots with buds from three individual alfalfa plants of the same growth state were selected for cuttings. The stems were cut into 8–10 cm segments and inserted into the nutrition soil containing peat, vermiculite and perlite (V:V:V = 1:1:1), each branch has one or two nodes and three to five leaves. Samples were taken at the base of 5 mm of stem segment during the four stages of alfalfa cutting according to the method of Brinker, Zyl, Liu, Craig, Sederoff, Clapham and Arnold [30] with modifications. All samples were immediately immersed in liquid nitrogen and stored at -80 ℃ for further experiments.

### Histology analysis

Paraffin transverse and Paraffin longitudinal sections of the stem base of alfalfa were made as described of Yuan, et al. [86] with modifications. Then they were stained with ferro red and green fixation to observe the cell morphological structure of the stem base of alfalfa under light microscope (NIKON, Japan).

### Preparation of libraries and Illumina sequencing

Total RNA was extracted from each sample and then treated with gDNA Eraser (TaKaRa, Japan) to remove contaminated genomic DNA. RNA degradation and contamination were monitored on 1% agarose gels and RNA Purity was checked using a NanoPhotometer spectrophotometer (IMPLEN, USA). RNA concentration was measured using a Qubit RNA Assay Kit in a Qubit 2.0 Fluorometer (Life Technologies, USA), and RNA integrity was assessed using the RNA Nano 6000 Assay Kit of the Bioanalyzer 2100 system (Agilent Technologies, USA). A total amount of 3 μg RNA per sample was used to construct a sequencing library [87]. Sequencing libraries were generated using the VAHTS ™ Universal DNA Library Prep Kit for Illumina ® V2 (NuoWeiZan, China) following the instruction manual, and index codes were added to attribute sequences to each sample. The Agilent Biological Analyzer 2100 system was used to detect the quality of Illumina libraries, and the library was sequenced by a Illumina NovaSeq 6000 instrument. After removing reads containing joint sequences and filtering low-quality reads from raw data, clean data were obtained and used for transcriptome analysis. Then the Clean Data were compared with alfalfa reference genome (https://figshare.com/s/fb4ba8e0b871007a9e6c) with the following parameters: adjusted *p*-value < 0.01 and |log_2_ FC|> = 2 on BMKCloud platform. And the DESeq2 software was used to analyze DEGs [88].

### Quantitative RT–PCR

To verify the results of DEG analysis, 17 genes with differential expression were selected and detected by qRT-PCR according to the method of Li, et al. [89]. The relative expression level of each gene was calculated using the 2 ^−∆∆Ct^ method [90]. Actin gene of alfalfa was used as internal reference gene, and the reference sequence was uploaded (Table S1).

### Function annotation

The unigene sequences were aligned to databases, including the Nonredundant protein (NR), SwissProt, Pfam, Gene Ontology (GO), Cluster of Orthologous Groups (COG), Kyoto Encyclopedia of Genes and Genomes (KEGG) [91], Evolutionary Genealogy of Genes: Non-supervised Orthologous Groups Database (eggNOG) and Cluster of Orthologous Groups (KOG/COG) databases [92]. GO annotation was performed by using Blast2GO software (version 2.3.5) under a threshold E-value ≤ 10^−5^. The KEGG Automatic Annotation Server (KAAS1) was used to obtain the KEGG pathway annotation [93–95], and HMMER software (Eddy 1998) was used to search the Pfam database.

### Assays of TFs and hormone-related DEGs

A comprehensive plant TF database was downloaded from http://planttfdb.cbi.pku.edu.cn/index.php [96], and all sequences of DEGs were compared with the sequence of the plant TF database using Blastx under an E-value ≤ 10^−5^. The Arabidopsis Hormone Database (AHD) was downloaded from http://ahd.cbi.pku.edu.cn/. To analyze the plant hormone-related DEGs, all DEGs from the Y-vs-C (Y versus C, C is control, the same is true for other comparisons), P-vs-Y groups were compared with protein sequences from AHD using Blastx (E-value ≤ 10^−5^).

### Function explore of candidate genes

Three DEGs, MsG0780035932.01, MsG0780040671.01 and MsG0380016110.01 identified in the Y-vs-C group were used as candidates to analyze their function in the AR formation. MsG0780035932.01, with a 498 bp CDS, encodes a small auxin-up RNA (SAUR). MsG0780040671.01, with a 1443 bp CDS, encodes an adenosine diphosphate (ADP)-ribosylation factor-guanosine triphosphatase (GTPase)-activating protein (ARF-GAP). And MsG0380016110.01, with a 1362 bp CDS, encodes glucan endo-1, 3-beta-glucosidase 4 (EGLC). PCR was carried out to clone the full length of these genes (Fig. S1), which were ligased to the plant expression vector 3302Y contain 35 s promoter and transferred into Agrobacterium EHA105, respectively. When the OD value reached 0.6–0.8, the agrobacterium solution was centrifuged, and MS solution was used to re-suspend the precipitated Agrobacterium. Six 8–10 cm branches of alfalfa with two buds were infected with each Agrobacterium. The lower part of alfalfa branchs was immersed in the infecting solution containing acetoeugenone for 20 min for instantaneous transformation. After the transformation, they were inserted separately into clear pots containing nutrient soil, which were sheltered from light with tinfoil, so that the development of ARs could be observed daily. Water, EHA105 and no load were used as control group 1, 2, 3, and MsG0780035932.01, MsG0780040671.01 and MsG0380016110.01 genes were used as experimental group 4, 5, 6, respectively. The bases of each cutting were observed and recorded every day.

### Subcellular localization of selected three DEGs proteins

Tobacco seeds were grown in the same way as those of alfalfa. MsG0780035932.01, MsG0780040671.01 and MsG0380016110.01 was linked to 3302Y vector containing YFP gene and transferred to Agrobacterium EHA105, respectively. When tobacco grew to 5–6 weeks, three genes was transformed into tobacco leaves by agrobacterium-mediated transient transformation, respectively. After two days of dark culture, subcellular localization was observed under the YFP channel of confocal microscope.

## Supplementary Information

**Additional file 1: Table S1.** Primers sequence used in the experiment. **Table S2.** Sequence alignment of sample sequencing data with the reference genome. **Table S3.** Number of DEGs annotated in RNA-Seq. **Table S4.** Number of DEGs involved in plant hormones.

**Additional file 2: Table S5.** List of annotated hormone related DEGs up-regulated or down-regulated in induction stage verse initial separation stage.

**Additional file 3: Table S6.** List of annotated hormone related DEGs up-regulated or down-regulated in AR primordium formation stage verse induction stage.

**Additional file 4: Figure S1.** Full length of MsG0780035932.01, MsG0380016110.01 and MsG0780040671.01 genes. **Figure S2.** Circuit diagram of Plant hormone signal transduction, including auxin, abscisic acid, brassinosteroid, cytokinin, ethylene, gibberellin, jasmonic acid and salicylic acid.

## Data Availability

The original data obtained from transcriptome sequencing have been uploaded to NCBI database at the login number PRJNA901129: https://www.ncbi.nlm.nih.gov/sra/PRJNA901129.
