# Supplementary material for: Uncovering early transcriptional regulation during adventitious root formation in Medicago sativa
Source: BMC Plant Biol. 2023 Apr 4;23:176. doi: 10.1186/s12870-023-04168-0 (PMC10074720; doi:10.1186/s12870-023-04168-0)
Supplement: Supplementary file 1 — Additional file 1: Table S1. Primers sequence used in the experiment. Table S2. Sequence alignment of sample sequencing data with the reference genome. Table S3. Number of DEGs annotated in RNA-Seq. Table S4. Number of DEGs involved in plant hormones. [file 12870_2023_4168_MOESM1_ESM.pdf]

Table S1 Primers sequence used in the experiment

primer	sequence
P-MsG0780035932.01-f	5'-ATGGATATTGGCAAGAACATGA -3'
P-MsG0780035932.01-r	5’-TTAGAAGTTGTTTAAGACAATC-3'
P-MsG0780040671.01-f	5'-CAAAAGAACTAGCTCGCCCC-3'
P-MsG0780040671.01-r	5'-GCATCTGAGTACTTGATATGGATGA-3'
P-MsG0380016110.01-f	5'-CCTCTCTTTCTTTGGATTAG-3'
P-MsG0380016110.01-r	5'-ACTATACAGCAATCCTAAACACCA-3'
MsG0780035932.01-3302Y-f	5'-cgggggactcttgacATGGATATTGGCAAGAACATGA-3'
MsG0780035932.01-3302Y-r	5'-actagtcagatctacTTAGAAGTTGTTTAAGACAATC-3'
MsG0780040671.01-3302Y-f	5'-cgggggactcttgacCAAAAGAACTAGCTCGCCCC-3'
MsG0780040671.01-3302Y-r	5'-actagtcagatctacGCATCTGAGTACTTGATATGGATGA-3'
MsG0380016110.01-3302Y-f	5'-CgggggactcttgacCCTCTCTTTCTTTGGATTAG-3'
MsG0380016110.01-3302Y-r	5'-actagtcagatctacACTATACAGCAATCCTAAACACCA-3'
3302Y-f	5'-TGACGCACAATCCCACTATCCTT-3'
3302Y-r	5'-CCGTCCAGCTCGACCAGGAT-3'
Ms-Actin-f	5'-GCTGTGGTTGCTTTTTTGGTGTCTC-3'
Ms-Actin-r	5'-TGGGCTGCCACAGAACATTTGA-3'
RT-MsG0880047188.01-f	5'-TGCTGGTAGATACAAAGC-3'
RT-MsG0880047188.01-r	5'-ATTCTCACAAGTAGTGGC-3'
RT-MsG0780038753.01-f	5'-TTTCTATGGTGGTAGTGATG-3'
RT-MsG0780038753.01-r	5'-TGTGCTTAGTGCTGCTGT-3'
RT-MsG0780041317.01-f	5'-GCATCTTCGGATCAAACG-3'
RT-MsG0780041317.01-r	5'-TCACCAACCCAACCCTTT-3'
RT-MsG0880043618.01-f	5'-GCCACTAGAACAAGAAGT-3'
RT-MsG0880043618.01-r	5'-TCACAGTAGCCAAGTAAG-3'
RT-MsG0680031607.01-f	5'-CAACTAAGCGGTGGCAGTA-3'
RT-MsG0680031607.01-r	5'-TGAAGACGAACCCGAAAA-3'
RT-MsG0780035932.01-f	5'-GTTATAGCTGCCAAGGAC-3'
RT-MsG0780035932.01-r	5'-AGAACGAGAACGACCAAA-3'
RT-MsG0780040671.01-f	5'-GGAGAAAGGAATGGGAGGTA-3'
RT-MsG0780040671.01-r	5'-ACGGGTGCGTTTGAGTAA-3'
RT-MsG0380016110.01-f	5'-ACAGTGCCTTAGTTGCTT-3'
RT-MsG0380016110.01-r	5'-CTGGAGGACTTGGTGAAT-3'
RT-Medicago_sativa_newGene_6090-f	5'-AGCGTCTCAGAATAGGCA-3'
RT-Medicago_sativa_newGene_6090-r	5'-GGAGTCAACTTTCGGCAC-3'
RT-MsG0480022309.01-f	5'-TCCCTTTGGACCTTACTG-3'
RT-MsG0480022309.01-r	5'-AACCTTACGGATTTCTCG-3'
RT-MsG0580027219.01-f	5'-TCGTTTGAGCAGGGTTGT-3'
RT-MsG0580027219.01-r	5'-TTGATCTAGGCGAGCCAC-3'

RT-MsG0780038793.01-f	5'-TTTCCAGGACCTCGTTTA-3'
RT-MsG0780038793.01-r	5'-GCCTTATTCCATGCCAAT-3'
RT-MsG0180002098.01-f	5'-AAGCAGAAGCAAAGAGGGTG-3'
RT-MsG0180002098.01-r	5'-AAGGTGGTGACAGAAGATGAAC-3'
RT-MsG0680035708.01-f	5'-CCACCAATCCGTTCTTTC-3'
RT-MsG0680035708.01-r	5'-GGGCACCATCCATGCTAA-3'
RT-Medicago_sativa_newGene_1972-f	5'-GGGGACAATCTTCTCACT-3'
RT-Medicago_sativa_newGene_1972-r	5'-TCTAACCAATGCCCTTTT-3'
RT-MsG0580025558.01-f	5'-CGGTGATAACGGTGGTGA-3'
RT-MsG0580025558.01-r	5'-TTCGGTGACAGACGCAAG-3'
RT-MsG0080048230.01-f	5'-GAGGTGGAGAAGTATGGG-3'
RT-MsG0080048230.01-r	5'-GTCCTAACCGTGGCATCA-3'

Table S2 Sequence alignment of sample sequencing data with the reference genome

Sample Name	Total Reads	Mapped Reads	Uniqn Mapped Reads	Multiple Map Reads	Reads Map to '+'	Reads Map to '-'
C1	45,057,618	35,613,547 (79.04%)	33,606,543 (74.59%)	2,007,004 (4.45%)	19,178,929 (42.57%)	19,207,896 (42.63%)
C2	46,205,968	35,142,028 (76.06%)	33,135,272 (71.71%)	2,006,756 (4.34%)	18,975,841 (41.07%)	19,022,615 (41.17%)
C3	48,095,460	37,587,750 (78.15%)	35,555,961 (73.93%)	2,031,789 (4.22%)	20,205,790 (42.01%)	20,197,316 (41.99%)
Y1	42,259,542	32,266,119 (76.35%)	30,253,289 (71.59%)	2,012,830 (4.76%)	17,511,162 (41.44%)	17,545,561 (41.52%)
Y2	43,506,762	33,542,369 (77.10%)	31,444,542 (72.28%)	2,097,827 (4.82%)	18,207,725 (41.85%)	18,236,198 (41.92%)
Y3	44,762,984	34,575,586 (77.24%)	32,407,836 (72.40%)	2,167,750 (4.84%)	18,793,221 (41.98%)	18,801,697 (42.00%)
P1	51,367,536	40,940,104 (79.70%)	38,278,168 (74.52%)	2,661,936 (5.18%)	22,315,602 (43.44%)	22,401,196 (43.61%)
P2	41,781,440	32,066,339 (76.75%)	30,104,995 (72.05%)	1,961,344 (4.69%)	17,360,073 (41.55%)	17,411,765 (41.67%)
P3	39,123,324	31,014,464 (79.27%)	29,043,642 (74.24%)	1,970,822 (5.04%)	16,871,639 (43.12%)	16,927,727 (43.27%)
S1	48,219,550	37,706,962 (78.20%)	35,354,256 (73.32%)	2,352,706 (4.88%)	20,481,984 (42.48%)	20,472,355 (42.46%)
S2	41,384,980	32,332,940 (78.13%)	30,266,799 (73.13%)	2,066,141 (4.99%)	17,569,444 (42.45%)	17,662,954 (42.68%)
S3	46,649,534	35,746,949 (76.63%)	33,440,713 (71.68%)	2,306,236 (4.94%)	19,493,914 (41.79%)	19,591,845 (42.00%)

Note: C1-C3, the stem cuttings isolated from the mature plant; Y1-Y3, the initial cell reprogramming; P1-P3, primordium formation at the base of the stem cutting; S1-S3, initiation of ARs and white bulges formation. Total Reads, the number of Clean Reads; Mapped Reads, number of Reads aligned to the reference genome and the percentage of the reads in Clean Reads; Uniq Mapped Reads, the number of Reads aligned to a unique position in the reference genome and the percentage of the reads in Clean Reads; Multiple Map Reads, the number of Reads matched to multiple locations in the reference genome and the percentage of the reads in Clean Reads; Reads Map to '+', the number of Reads aligned to the plus strand of reference genome and the percentage of the reads in Clean Reads; Reads Map to '-', the number of Reads aligned to the negative strand of reference genome and the percentage of the reads in Clean Reads.

Table S3 Number of DEGs annotated in RNA-Seq

Annotation Database	Y-vs-C	P-vs-Y	S-vs-P
COG	3291	2114	47
GO	7953	5449	127
KEGG	6504	4500	101
KOG	4666	3153	61
Pfam	7376	5095	130
Swiss-Prot	6875	4781	121
eggNOG	7779	5419	128
NR	9078	6357	150
All	9095	6371	150

Note: Y-vs-C: induction stage verse initial separation stage; P-vs-Y: AR primordium formation stage verse induction stage; S-vs-P: AR maturation stage verse AR primordium formation stage.

Table S4 Number of DEGs involved in plant hormones

plant Hormone	Y-vs-C		P-vs-Y	
	Up-regulated	Down-regulated	Up-regulated	Down-regulated
auxin	35	42	31	31
brassinosteoid	29	38	36	14
cytokinin	8	26	26	4
abscisic acid	21	28	25	13
ethylene	10	9	12	16
gibberellin	14	30	27	8
jasmonic acid	9	26	23	6
salicylic acid	11	14	19	6

Note: Y-vs-C: induction stage verse initial separation stage; P-vs-Y: AR primordium formation stage verse induction stage.
