# Supplementary material for: Uncovering early transcriptional regulation during adventitious root formation in Medicago sativa
Source: BMC Plant Biol. 2023 Apr 4;23:176. doi: 10.1186/s12870-023-04168-0 (PMC10074720; doi:10.1186/s12870-023-04168-0)
Supplement: Supplementary file 4 — Additional file 4: Figure S1. Full length of MsG0780035932.01, MsG0380016110.01 and MsG0780040671.01 genes. Figure S2. Circuit diagram of Plant hormone signal transduction, including auxin, abscisic acid, brassinosteroid, cytokinin, ethylene, gibberellin, jasmonic acid and salicylic acid. [file 12870_2023_4168_MOESM4_ESM.pdf]

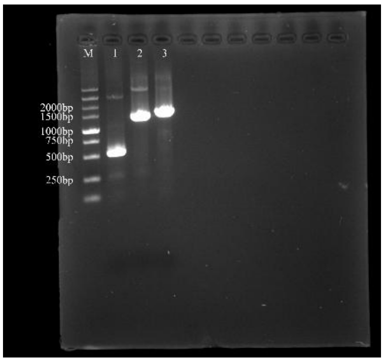

Figure S1. Full length of MsG0780035932.01, MsG0380016110.01 and MsG0780040671.01 genes. Note: M: DNA Marker DL5000; 1: Full length of MsG0780035932.01 gene; 2: Full length of MsG0380016110.01 gene; 3: Full length of MsG0780040671.01 gene. Only one exposure was made for the gel.

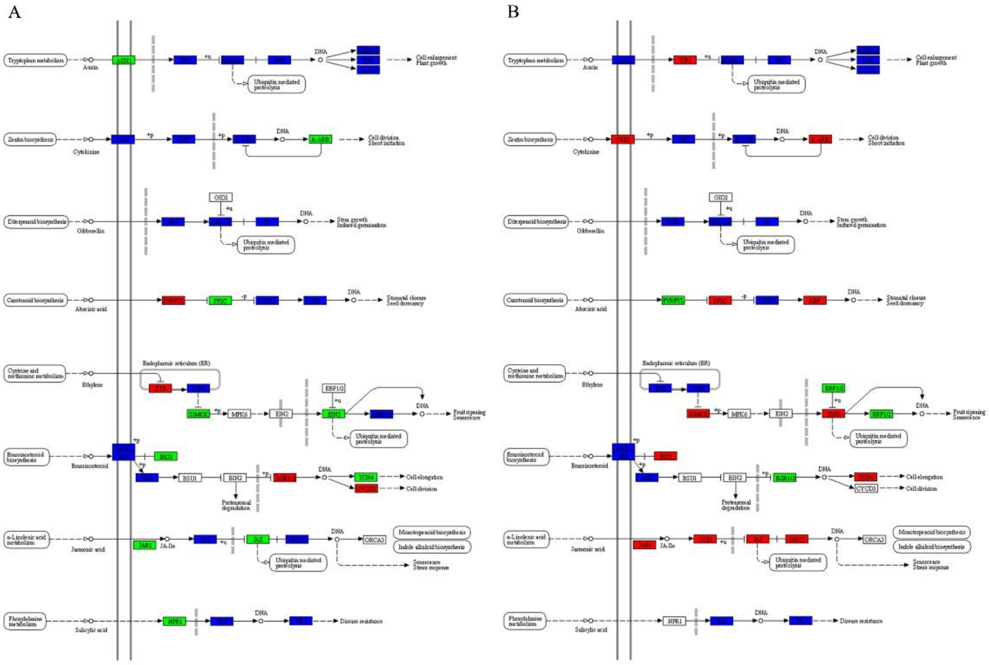

Figure S2. Circuit diagram of Plant hormone signal transduction, including auxin, abscisic acid, brassinosteroid, cytokinin, ethylene, gibberellin, jasmonic acid and salicylic acid. (**a**). Circuit diagram of Plant hormone signal transduction in induction stage verse initial separation stage. (**b**). Circuit diagram of Plant hormone signal transduction in AR primordium formation stage verse induction stage. Red represents up-regulated expression, green represents down-regulated expression and blue represents both up-regulated and down-regulated expression [1-3].

1 Kanehisa M, Goto S. KEGG: kyoto encyclopedia of genes and genomes. Nucleic Acids Res. 2000;28(1):27-30. doi:10.1093/nar/28.1.27.

2 Kanehisa M. Toward understanding the origin and evolution of cellular organisms. Protein Sci. 2019;28(11):1947-1951. doi:10.1002/pro.3715.

3 Kanehisa M, Furumichi M, Sato Y, Kawashima M, Ishiguro-Watanabe M. KEGG for taxonomy-based analysis of pathways and genomes. Nucleic Acids Res. 2023;51(D1):D587-d592. doi:10.1093/nar/gkac963.
